# A Lethal Complication after Transarterial Chemoembolization with Drug-Eluting Beads for Hepatocellular Carcinoma

**DOI:** 10.1155/2015/873601

**Published:** 2015-02-23

**Authors:** Adriana Toro, Gaetano Bertino, Maria Concetta Arcerito, Maurizio Mannnino, Annalisa Ardiri, Domenico Patane', Isidoro Di Carlo

**Affiliations:** ^1^Department of Surgical Sciences, Organ Transplantation and Advanced Technologies, University of Catania, Cannizzaro Hospital, 95100 Catania, Italy; ^2^Department of Internal Medicine and Systemic Disease, Hepatology Unit, University of Catania, S. Marta Hospital, 95100 Catania, Italy; ^3^Department of Emergency, Vittorio Emanuele Hospital, 93012 Gela, Italy; ^4^Department of Radiology, Cannizzaro Hospital, 95100 Catania, Italy

## Abstract

*Background*. The current standard of care for patients with large or multinodular noninvasive hepatocellular carcinoma is conventional transarterial chemoembolization (TACE). TACE may also be performed with drug-eluting beads, but serious complications of this procedure have been reported. *Methods*. Aim of this report is to present a patient affected by multifocal HCC who underwent TACE with drug-eluting bead (DEB-TACE). *Results*. Following the procedure the patient developed a hepatic abscess and biliobronchial fistula resulting in adult respiratory distress syndrome and death. *Conclusion*. We speculate that DEB-TACE has a prolonged effect on the tumor and the surrounding liver, resulting in progressive enlargement of the necrotic area. This activity that can extend to the surrounding healthy hepatic tissues may continue indefinitely.

## 1. Introduction

The current standard of care for patients with good liver function who have large or multinodular noninvasive hepatocellular carcinoma (HCC) that is not suitable for surgical resection or radio frequency ablation (RFA) is transarterial chemoembolization (TACE) able to improve survival.

According to different authors transarterial chemoembolization is indicated for three or less lesions or for five or less lesions. In addition many authors do not recommend single transarterial chemoembolization for lesion larger 8.0 or 10.0 cm due to the association of significant morbidity. This standard is based on the results of a meta-analysis of randomized controlled trials [1]. Conventional TACE involves infusion of an anticancer-in-oil emulsion followed by embolic agents. More recently, TACE has also been performed with embolic drug-eluting beads (DEB-TACE). DEB are soft, deformable, spherical-shaped beads made of polyvinyl alcohol and a hydrophilic ionic monomer that can be loaded with an anthracycline drug such as doxorubicin [2]. The beads are loaded* in vitro* before the procedure and gradually release the chemotherapeutic agent after delivery into the tumor [2]. This technique minimizes the systemic release of drugs and thereby minimizes systemic effects. As with conventional TACE, postembolization syndrome (PES) is the most frequently reported complication. PES is usually self-limiting, but some potentially fatal complications of DEB-TACE have also been reported [3]. This report presents a patient who underwent DEB-TACE and developed a fatal complication, and includes a review of the literature regarding this complication.

## 2. Case Report

A 64-year-old Caucasian man was referred to us in February 2011 after abdominal computed tomography (CT) that showed three nodules in his liver, suggestive of HCC.

**Figure 1:**
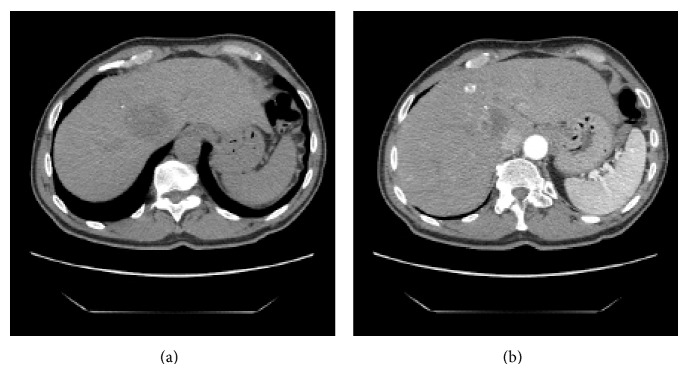
Pretreatment CT scan (a) CT image showing the main tumor in the right lobe of the liver at the confluence of the right and middle hepatic veins measuring 42 × 35 mm. (b) CT-image in the arterial phase showed the second lesion in segment 4 measuring 12 mm in diameter.

The patient had been diagnosed with liver cirrhosis secondary to hepatitis B virus infection in 1999, based on liver biopsy findings. He had a history of hiatus hernia, right-sided nephrolithiasis, lumbar intervertebral disc herniation, and prostatic hypertrophy, all of which were treated medically. The patient was performance status ECOG 2. He was followed up every 6 mo by hepatologists. Ultrasonography at his last visit showed three masses in the liver which were suspicious for HCC. Abdominal CT showed three hypervascular lesions in the liver: one in segment 8, at the confluence of the right and middle hepatic veins, measuring 42 mm × 35 mm (Figure 1(a)), one in segment 4 measuring 12 mm in diameter (Figure 1(b)), and one between segments 6 and 7 measuring 25 mm in diameter. His serum alpha-fetoprotein level was normal (3.21 ng/mL, value normal 0–7 ng/mL). He had Child-Pugh class A liver function, with normal liver function tests. His indocyanine green retention rate at 15 min was measured to assess his hepatic reserve and plan surgical resection. According to the Makuuchi algorithm [4], his retention rate of 14% indicated that he should not undergo major liver resection (more than three segments). As he did not meet the criteria for curative resection or radiofrequency ablation, DEB-TACE was planned.

DEB-TACE was performed as follows. Peri-interventional management included complete cardiopulmonary monitoring (electrocardiography, blood pressure monitoring, and oxygen saturation monitoring), analgesic sedation, antiemetic prophylaxis, and antibiotic prophylaxis (metronidazole and a cephalosporin). Before of the embolization, angiography showed 3 blushed hypervascular lesions. The portal vein was patent. A drug-loading solution was prepared by adding sterile water to a vial of doxorubicin hydrochloride powder. The saline solution was removed from the beads and the doxorubicin solution was added and left for a time until the beads had taken on a red color. Loaded beads were aspirated into a syringe and mixed with a nonionic contrast medium.

A microcatheter was positioned in the right hepatic artery and was injected with an emulsion of DEB (300–500 *μ*m; Terumo DC-Bead Italia SRL, Rome, Italy) loaded with doxorubicin 50 mg. A small amount of the emulsion was also injected into the left hepatic artery for embolize the lesion of the segment 4. Clinical examinations were performed within 24 h after the procedure and before discharge. Follow-up contrast-enhanced multidetector-row CT was performed 1 and 3 mo after DEB-TACE showing necrosis of all the lesions. Tumor response was assessed after 3 mo using the modified Response Evaluation Criteria in Solid Tumors (mRECIST) with which a complete response was defined as disappearance of intratumoral arterial enhancement in all lesions, a partial response was defined as a 30% decrease in the sum of the diameters of viable (contrast-enhanced in the arterial phase) lesions, progressive disease was defined as an increase of 20% in the sum of the diameters of viable lesions, and stable disease was defined as remaining disease that did not fit the criteria for a partial response or progressive disease. The objective response rate was defined as sum of the complete and partial response rates. In our patient tumor response assessed after 3 mo according to the mRECIST showed complete response with the disappearance of any intratumoral arterial enhancement in all target lesions [5].

The procedure was performed in March 2011. The patient did not develop postembolization syndrome (abdominal pain, nausea, vomiting, or fever) and was discharged the day after.

**Figure 2:**
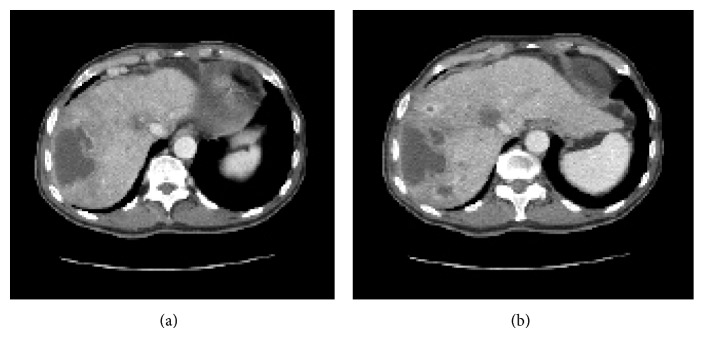
CT image at 28 d after TACE with DC beads. (a) CT image showing a huge abscess in the right lobe of the liver. (b) Many small hypodense areas are located mostly in segments 4, 6, 7, and 8 of the liver.

Twenty-eight days after the procedure, he returned to our department with right upper quadrant abdominal pain and fever (39°C). Abdominal CT showed a large hypodense area in the right lobe of the liver measuring 78 mm × 82 mm (Figure 2(a)) and multiple small hypodense areas throughout the right lobe, especially in segments 4, 6, 7, and 8 (Figure 2(b)). He was admitted to hospital, and a CT-guidance percutaneous 10-Fr drainage catheter was placed in the large hepatic abscess. Antibiotics (metronidazole plus tazobactam) and paracetamol were administered. His fever and pain resolved within 48 h. As the catheter continued to drain a small amount of purulent necrotic material, it was left in place after discharge and he was seen daily in the output clinic. The drainage stopped after 2 wk, and after repeat ultrasonography the catheter was removed.

The patient remained asymptomatic for 10 d and then represented with right upper quadrant abdominal pain and fever. He was admitted to hospital, and was treated with the same antibiotic regimen as previously. Abdominal CT showed a fluid collection at the location of the previous large abscess. Another percutaneous drainage catheter was placed, and 100 mL of grossly purulent material was aspirated. The organisms isolated from the aspirated material were sensitive to the antibiotics he was receiving. The drainage catheter was left in place while drainage of necrotic material continued. After almost 3 wk, when he was asymptomatic and the drainage had stopped, the catheter was removed.

**Figure 3:**
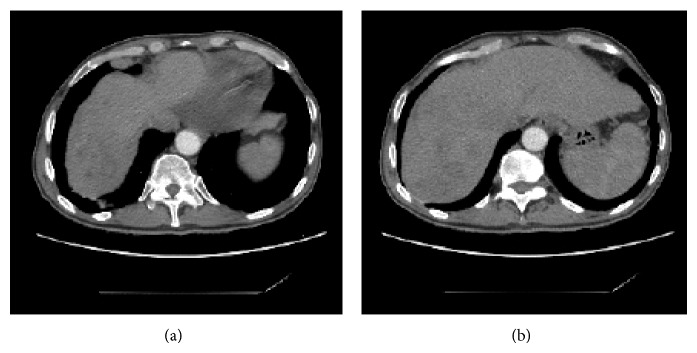
CT scan before presenting to our emergency room with bilioptysis and fever. (a) There is a huge fluid collection adjacent to the dome of the right lobe of the liver. (b) Multiple abscesses are scattered throughout the right lobe of the liver.

The patient was then lost to follow-up for 3 mo before presenting to our emergency room with bilioptysis and fever (39°C). Abdominal CT showed a huge fluid collection (Figure 3(a)) in the right lobe of the liver and a narrow communication between the collection and the thoracic cavity. There were multiple abscesses throughout the right lobe of the liver (Figure 3(b)). A biliobronchial fistula was diagnosed by bronchoscopy. The patient died of adult respiratory distress syndrome 2 wk later.

## 3. Discussion

The viability of HCC depends on its arterial supply. TACE with iodized oil and embolic particles interrupts the arterial supply to the tumor, causing ischemia. The iodized oil also causes temporary obstruction of the portal venules in the peribiliary plexus. Some studies reported that ischemia played a role in the effective treatment of HCC by TACE when only a small dose of drug was delivered [6]. A pharmacokinetic study found that TACE with iodized oil increased the concentration and half-life of doxorubicin in the tumor and lowered the peak concentration of the chemotherapeutic agent in the systemic circulation. Use of lipiodol results in a higher deposition of adriamycin at the tumor site than in the adjacent hepatic parenchyma.

DEB-TACE is a procedure that uses microspheres as embolic material which are loaded with doxorubicin and ensure a slow and uniform release in the lesion-target: this allows a greater local concentration of drug with lower peak plasma concentration compared to TACE, reducing the effects systemic toxic. These microspheres are made using a unique drug-eluting bead technology and are composed of polyvinyl alcohol hydrogel that has been modified with sulfonate groups [2]. Doxorubicin is a chemotherapeutic anthracycline glycolide agent that is widely used for the treatment of HCC and can be loaded into DEB [2]. Delivery of the beads into the feeding vessels of the tumor causes lumen occlusion and ischemia and continued local release of doxorubicin leads to tumor necrosis [7]. This technique has the advantage of resulting in a lower plasma concentration of doxorubicin than conventional intra-arterial administration. The concentration of doxorubicin in the tumor peaks on day 3, remains high for 7 d, and starts to decline after 14 d [7]. Tumor necrosis is greatest at 7–14 d. During this period, the proportion of damaged and necrotic cells in the tumor approaches 100%, and the plasma doxorubicin concentration is minimal.

A study using a Vx-2 rabbit model with intra-arterial infusion found that the period of doxorubicin release ranged from 60 to 100 d and that the intratumoral doxorubicin level at 72 h after embolization was about four times higher than after conventional TACE [7]. Histological examination showed that the areas of necrosis were centered around clusters of DEB, with extensive liquefactive and coagulative necrosis suggesting cell death resulting from both ischemic and toxic causes [8]. High treatment response rates can be achieved using small beads (100–300 or 300–500 *μ*m). Small beads can achieve distal embolization and obstruct collateral vessels, resulting in pan-necrosis of the target lesion. Smaller DEB can penetrate further into targeted tissue with a higher spatial density with greater and more uniform drug coverage. However, use of small beads can also result in ischemic necrosis of the adjacent liver [8]. For some authors the use of DEB-TACE in patients for liver transplantation for HCC can increase recurrence-free survival after liver transplantation; however in patients treated with conventional TACE was not observed an intense inflammatory and fibrotic reaction in the area surrounding the tumor tissue that was observed in native liver treated with DEB-TACE [9].

Studies phase I/II and cohort studies have shown promising efficacy and low toxicity. However, there is currently no evidence of a benefit in terms of survival in the use of DEB-TACE compared to TACE: recent studies of phase II reported survival rates at 1 and 5 years essentially comparable with those reported with TACE [10]. The available studies directly comparing the two methods have produced conflicting results. A small study documents increased survival of patients undergoing DEB-TACE compared to those treated with TACE and another greater necrosis after DEB-TACE occurred on the explanted livers. The European multicenter randomized “PRECISION V” study detected that the DEB-TACE is more effective than TACE in terms of disease control and tolerability, especially in certain subgroups of patients more vulnerable as Child-Pugh class B, bilobar disease, and relapsed disease [11]. The DEB-TACE has the advantage of being more standardized methodology, and it presents more rarely postembolization syndrome, which is of lesser severity.

A randomized study did not demonstrate any advantage between TACE and DEB-TACE in terms of tumor recurrence and survival at six months [12]. A retrospective cohort study demonstrated improved survival after TACE (median 46 mo to 19 mo to DEB-TACE: *P* < 0.0001) and a prolonged time to progression (30 mo versus 16 mo to DEB-TACE: *P* = 0.003), with no differences in toxicity [13]. Finally a meta-analysis showed that in HCC patients who are not suitable for other curative treatments the DEB-TACE is a better choice, but none randomization study was analyzed, none study analyzed the overall survival, and different criteria to evaluate tumor response were used to led to different interpretation results [14].

This could represents a valid indications of  TACE as initial treatment, in patients with tumors exceeding 50 mm in order to prevent the risk of complications of DEB-TACE and to permit the shrinkage of the mass. This treatment is valid especially if the tumor is not so large and if the definitive treatment as resection or transplantation cannot be advisable also after initial treatment with TACE or DEB-TACE.

The main risk factors for complications after TACE include main portal vein obstruction, compromised hepatic functional reserve, biliary obstruction, previous biliary surgery, excessive lipiodol injection, and nonselective embolization. PES is a complication uniquely associated with TACE, not only related to the liver tumors procedures, but also observed in the case of embolization of uterine fibroids or preoperative tumor embolization. Even though it is a self-limiting condition, PES prolongs hospitalization and postpones additional treatment. PES commonly presents with fever, nausea, vomiting, right upper quadrant abdominal pain, and elevated serum transaminase levels. The precise mechanisms underlying PES are not clear, but proposed hypotheses include acute ischemia of the liver parenchyma, distension of the liver capsule, and gallbladder ischemia following cystic artery embolization [15]. Treatment is mainly supportive with antiemetic, analgesic, and antipyretic medication and steroids in severe cases.

In contrast, liver abscess and cholecystitis are considered to be severe adverse events. The reported rate of abscess formation after DEB-TACE ranges from 1.4% to 7.4% [9], and after conventional TACE is 0.2%. Signs of liver abscess include morning fever, leukocytosis, pain, and chills presenting at least 4-5 d after treatment. Abscess formation is attributed to lodgment of circulating bacteria in the necrotic liver.

Abscess formation has been reported in patients with a patent portal vein and no history of bilioenteric anastomosis (which is major risk factor for this complication), even after antibiotic prophylaxis with cephalosporin and metronidazole. It was reported that an aggressive prophylactic regimen including 24–36 h of intravenous tazobactam/piperacillin administered on an inpatient basis, plus bowel preparation with neomycin, erythromycin, and bisacodyl sodium, reduced abscess formation after TACE [11]. However, this needs further evaluation as another study found that this regimen did not result in a significant reduction in abscess formation.

It has been suggested that the relatively high rate of abscess formation after TACE with doxorubicin-eluting beads may be caused by prolonged exposure to the beads, which are not absorbed [3]. Hepatic abscesses can be usually treated with antibiotics, rehydration, analgesic, and antipyretic drugs, and percutaneous drainage. Most patients recover, but several fatal cases have been reported [3]. Other severe and potentially fatal complications after DEB-TACE have also been reported. As in our case, the other reported deaths resulted from complications associated with abscess formation.

A biliobronchial fistula (BBF) is an abnormal communication between the bile ducts and the bronchi and can be congenital, posttraumatic, iatrogenic, or secondary to infection [16]. Two major factors are considered to be responsible for the development of BBF: the mechanical effect of an increased intraluminal pressure of the biliary system due to bile duct obstruction and the local inflammatory or infectious process such as a subphrenic or intrahepatic abscess, thus triggering the adhesion between the lung and the diaphragm. Close contact between the hepatic dome and the diaphragm, the thoracoabdominal pressure gradient, ischemia, inflammation of the diaphragm, and the corrosiveness nature of bile may contribute to the formation of a biliobronchial fistula.

In the present case, the formation of the subphrenic liver abscess was an effect of DEB-TACE and its growth can be considered the cause of intrahepatic bile ducts obstruction. The infection in association with the corrosiveness of the bile can have led to rupture of the abscess into the pleural space, creating consequently a BBF. Patients may present with various respiratory and digestive symptoms, but bilioptysis with a long history of chronic irritable cough is pathognomonic for this condition. Diagnosis is based on chest radiography, ultrasonography, CT, and magnetic resonance imaging findings. A direct evidence can be provided by endoscopic retrograde cholangiopancreatography (ERCP) or percutaneous transhepatic cholangiography (PTC). In cases that are difficult to diagnose, a hepatoiminodiacetic acid scan may be helpful. Bronchoscopy is useful for evaluating bronchial involvement.

The treatment for BBF is demanding. The somatostatin and its analogues reducing the gastrointestinal secretions can be used for treating BBF. At present no author has report a case of the BBF treated with only medical therapy [17]. The options have been traditionally surgical: simple drainage of the subphrenic abscess with or without resection of the fistulous tract with hepatic resection and of involved lung. However, the operative procedures for BBF are frequently complex with significant morbidity and mortality.

In a previously reported case [18] with similar features to our case (except for the biliobronchial fistula), the patient was initially treated with percutaneous drainage and underwent right hemihepatectomy 5 mo later. The patient developed adult respiratory distress syndrome and died 25 d after surgery. Histopathological examination of the resected liver specimen showed HCC with a large area of necrosis surrounded by poorly differentiated small cells [18].

## 4. Conclusion

We speculate that DEB-TACE has a prolonged effect on the tumor and the surrounding liver, resulting in progressive enlargement of the necrotic area. The area of necrosis may therefore extend beyond the tumor into the surrounding healthy hepatic tissues. As DEB are not readily absorbed, this effect may continue indefinitely.

## Abbreviations

HCC: Hepatocellular carcinoma
RFA: Radio frequency ablation
TACE: Transarterial chemoembolization
DEB: Drug-eluting bead
CT: Computed tomography
mRECIST: modified Response Evaluation Criteria In Solid Tumor
PES: Postembolization syndrome
BBF: Bibliobronchial fistula.
